# Delayed Management of an Orbital Floor Blow-out Fracture

**DOI:** 10.14744/bej.2021.94834

**Published:** 2021-09-27

**Authors:** Aida Pidro, Nina Jovanovic, Emina Kadribašic, Nedim Barucija, Nedim Leto, Alon Kahana

**Affiliations:** 1. Department of Ophthalmology At Opca Bolnica “Prim. Dr. Abdulah Nakas”, Sarajevo, Bosnia and Herzegovina; 2. Department of Ophthalmology At Canton Hospital Zenica, Bosnia and Herzegovina; 3. Department of Maxillofacial Surgery, Canton Hospital Zenica, Bosnia and Herzegovina; 4. Department of Ophthalmology, Oakland University William Beaumont School of Medicine, Michigan, USA

**Keywords:** Enophthalmos, hypoglobus, orbital fracture, transconjunctival approach

## Abstract

A bony fracture in the orbital floor, the most common site, can lead to tissue herniation, enophthalmos, hypoglobus, or strabismic diplopia. Several surgical approaches for repair have been described in the literature. This report is a description of an illustrative case and a brief summary of the literature related to the transconjunctival approach to orbital floor fracture repair as performed by ophthalmologists. A 19-year-old female patient had fallen from a 5-meter-high fence and sustained panfacial fractures, including both orbits and the surrounding sinuses. An acute repair was performed by a maxillofacial team to stabilize the facial structure . Following neurosurgical stabilization, she was referred to ophthalmology with pronounced hypoglobus and enophthalmos, diplopia, relative afferent pupillary defect, and a slightly pale right optic nerve head. Surgery was performed under general anesthesia using the transconjunctival approach and an alloplastic implant. This approach was effective, providing excellent exposure while reducing the risks of lower eyelid retraction and surgical scars associated with the transcutaneous approach.

## Introduction

The most common orbital fracture sites involve the inferior and medial walls along the thinnest bony areas. Fractures can be isolated or combined with other non-orbital fractures (1). They are more common in males, age 21–31, usually as a result of a fall, motor vehicle accident, or an assault (2). Pathognomonic clinical findings are diplopia, restricted eye movement, decreased periocular sensation, subcutaneous emphysema, and globe dystopia, typically but not always associated with chemosis and ecchymosis (3). In orbital “blow-out” fractures, the inferior orbital rim remains intact. In “blow-in” fractures, the inferior rim is fractured, typically as part of a zygomaticomaxillary complex fracture. The exam in this case reveals a step-off and point tenderness along the infraorbital rim. Due to acute edema and hemorrhage, exophthalmos can be present prior enophthalmos. As with any periocular trauma, the emergent exam must first establish that the globe is intact and that intraocular contents are undamaged (e.g., intraocular bleeding and retinal detachment) (3). The gold standard for assessing orbital trauma is a computed tomography (CT) scan without contrast (1). Surgical repair involves reduction of the fracture, with repositioning of herniating tissues back into the orbit, followed by stabilization with placement of an implant or graft along the fracture site. Emergent surgical repair is needed in cases involving extraocular muscle incarceration within a trapdoor fracture, acute enophthalmos, and/or hypoglobus (3). In other cases, observation is often recommended, to allow for resolution of edema before definitive assessment of the need for surgery. If needed semi-urgently, surgery is usually performed within 1–2 weeks of injury. Otherwise, it is recommended that surgery be delayed at least 3 months to allow tissues to heal before further intervention.

Surgical approach to the orbital floor can utilize a transcutaneous approach, typically a subciliary incision, or alternatively a transconjunctival approach with a fornix incision, in which case the scar is hidden. The aim of this report is to describe the use of the transconjunctival surgical approach as well as summarize and compare the two different approaches to orbital floor fracture repair.

## Case Report

**Figure 1.**
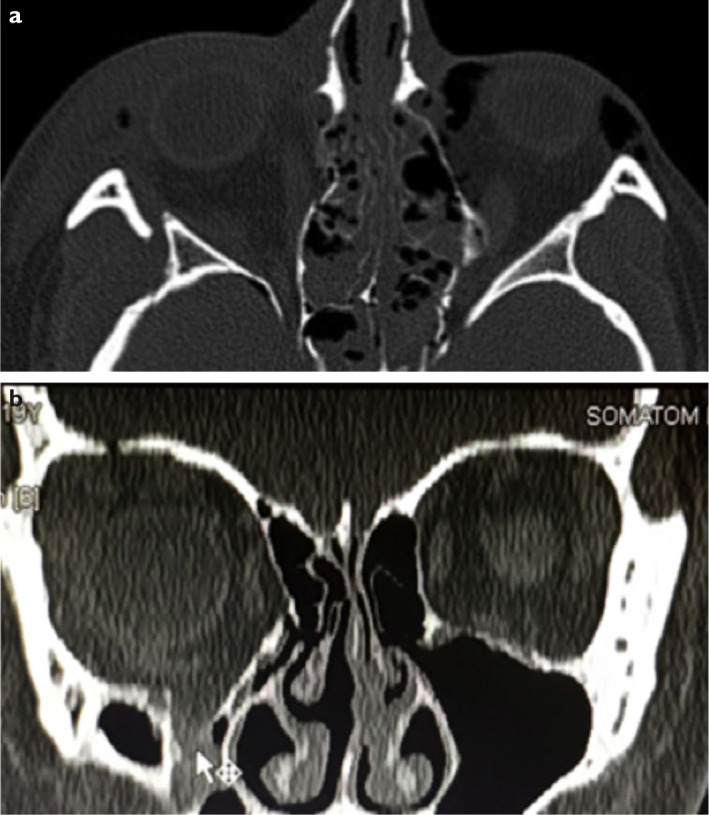
CT scans at the initial presentation showing. **(a)** Axial scan with lateral and medial wall fracture. **(b)** Coronal scan with floor and medial wall fractures.

A 19-year-old female presented at the ophthalmology department with marked hypoglobus and enophthalmos. Her medical history revealed a fall from a 5 m high fence on a concrete surface 7 months before presentation, when she sustained panfacial fractures to include both orbits and surrounding sinuses and had multiple surgical treatments thereafter. Immediate post-injury ophthalmology exams obtained at bedside while she was in induced coma revealed bilateral periorbital hematoma with edema, conjunctival chemosis, right pupil slightly more dilated, and the impalpable right-side orbital rim. CT scans had shown extensive fracture of both ethmoid and maxillary sinus walls, right orbital medial and inferior wall, with bony fragments dislocation, fat prolapse, and hemorrhagic content in the right maxillary sinus (Fig. 1a, b).

After vital functions were stabilized, the maxillofacial team (MFT) performed facial and orbital fractures surgical repair. The extended lateral canthal incision approach for the orbital roof repair was used, placing the osteosynthesis plate for fixation of the frontozygomatic suture region and the trans-oral approach for dislocated mandibular fracture, repositioning manually bone fragments in the opposite direction of the traumatic impression. Bone fragments were fixated with screws and osteosynthesis with titanium miniplate and interdental wire. Both maxillary sinuses were approached through their anterior walls and free bone fragments removed. The right-side hemorrhagic content was evacuated, orbital fat tissue partially repositioned in orbit through the roof of the sinus, and iodoform tamponade placed to stabilize the orbital floor from the inside of the maxillary sinus.

**Figure 2.**
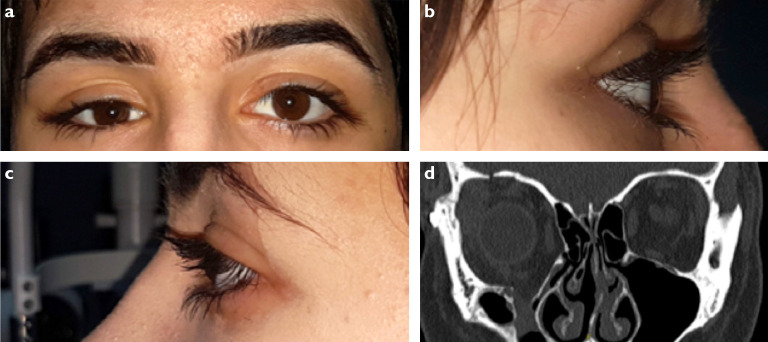
The clinical examination performed seven months after the initial surgery showing the **(a)** hypoblobus and enophthalmos, **(b)** right eye, **(c)** left eye **(d)** CT coronal scan with the floor and medial wall fracture.

Oculoplastic clinical examination, 7 months after the injuries and MFT management, revealed best-corrected visual acuity of 20/25 in both eyes, ocular normotension, with intact extraocular movement. There were relative hypoglobus and enophthalmos on the injured right side, with hypoglobus of 5 mm and enophthalmos of 4 mm by Hertel exophthalmometry (right eye 13 mm and left eye 17 mm) (Fig. 2a-C). There was also a right relative afferent pupillary defect (RAPD), with a slightly pale and decentered right optic nerve head. The patient’s chief complaints addressed her physical appearance and double vision in up gaze.

CT scan revealed an extension of the orbital fat tissue herniating into the maxillary sinus through the floor fracture. The fractured medial wall of the ethmoid sinus was indented inward by the orbital soft tissue. Both globes and other orbital structures were of regular form and size, with the right globe clearly enophthalmic on the scan (Fig. 2d).

**Figure 3.**
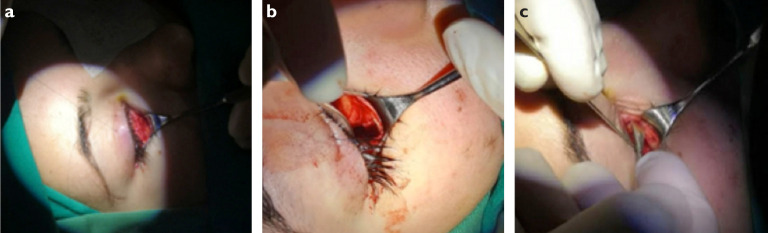
Surgical management via **(a)** Transconjunctival approach, **(b)** and **(c)** exposure of the orbital floor.

Surgically, the orbital floor was approached transconjunctivally through the inferior fornix, retracting the lower eyelid, protecting the globe and deepening the fornix with a ribbon retractor, and placing the incision 4–6 mm below the tarsus. A traction suture was placed to elevate the conjunctiva and retractors over the cornea, using 6–0 nylon suture (Fig. 3a). The dissection was advanced down to the infraorbital rim, elevating the periosteum posteriorly. Herniating orbital tissue was reduced from the fracture back into the orbital space (Fig. 3b, c). Special care was taken not to damage the infraorbital neurovascular bundle.

Exposing the orbital floor, the size of a fracture was measured to adjust the size and shape of a 0.35 mm thick nylon implant. Several small holes were punched through the implant to allow for tissue ingrowth and integration. Orbital tissues were allowed to lay onto the implant without the need for fixation. Forced duction test initially revealed persistent restriction, so the implant was removed and adjusted. Repeat forced duction test was negative. At that point, the nylon suture was released and the fornix incision closed by repairing the lower eyelid retractors with buried interrupted polyglactin suture.

**Figure 4.**
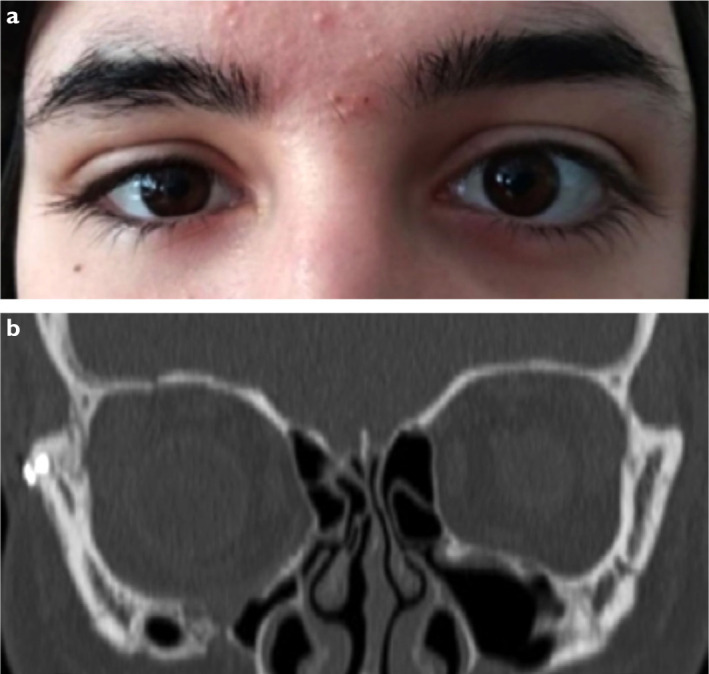
Patient at 14 weeks follow-up with improved **(a)** hypoglobus and enophthalmos **(b)** CT scan at 14 weeks follow up.

Post-operative treatment included systemic antibiotics, pain relief medication, topical ophthalmic antibiotic ointment, head elevation, and monitoring. The patient’s post-surgical recovery was unremarkable. At 1-week follow-up, the relative enophthalmos improved from 4 mm to 2 mm, hypoglobus was minimal (Fig. 4a, b), and no RAPD was shown. Long-term follow-up revealed persistent improvement of globe position. The patient did not complain of diplopia and was satisfied with the esthetic appearance.

## Discussion

The timing of orbital floor repair is always a matter of discussion. Most authors advise a 2-week delay to provide the swelling and accompanying diplopia to spontaneously resolve (4). Immediate repair is indicated if there is a large fracture (>50%), oculocardiac reflex, muscle entrapment with persistent restrictive strabismus and diplopia or significant hypoglobus or enophthalmos (>2 mm), and progressive infraorbital hypoesthesia (1,5). This delayed repair was performed due to residual hypoglobus, enophthalmos, RAPD, and double vision, after the initial traditional approach through the roof of maxillary sinus previously performed in MFS department. Delayed reconstruction leads to fibrosis, which could cause entrapped tissue to contract and restrict the globe motility (3). Even though surgically more challenging, late approach was still described as successful (6). Relative contraindications for surgery are hyphema, retinal tears, perforating globe injuries, or medical instability (7).

Approaches for orbital floor repair are varied. The subciliary approach commonly has a significant rate of complications, including scarring and eyelid retraction, when compared with the subtarsal and transconjunctival approach (12.9%, 1–3%, and <1%, retrospectively) (8). Importantly, the subciliary approach has a much higher rate of scleral show from eyelid retraction, with resultant dry eye issues as well as aesthetic concerns (9). While the subciliary and subtarsal approaches provide extensive exposure to the fracture site, the risks of an external scar and cicatricial retraction have made the transconjunctival approach the standard of care in much of the developed world. The transconjunctival exposure can be enhanced as needed through a lateral canthotomy and inferior cantholysis (“swinging eyelid approach”) (10).

The material used for reconstruction should be the one that allows for optimal stability, support, and the lowest risk of complications. Autografts were used more frequently in the past before alloplastics improved in biocompatibility and constitution (1). Autografts were golden standard for reconstruction due to their strength, vascularization, minimal inflammation and reactivity, and biocompatibility, but have an increased risk of complications due to inadequate malleability, unavailability, and unknown resorption (11). The size of an implant should not largely overextend the fracture area since it can cause the restriction in globe motility and position. However, it is important that the implant lay on bony ledges all around the fracture to reduce the risk of tissue herniation around the implant, which can lead to restrictive strabismus. There are several types of implants commonly in use, including metals (titanium and cobalt) and polymers. Titanium mesh is inert, corrosion resistant, tissue tolerant, and appropriate for large defects (11). Disadvantages include sharp edges, hard replacement, and high cost (12). Polyethylene porous implants are malleable, have a smooth surface, provide a good biocompatibility, and have lower infection rates (13). Silicone, nylon, and Teflon are non-porous and non-absorbable implants. Silicon is cheap, easy to work with but is known for frequent infections and extrusions (11). Teflon is non-antigenic and malleable, but is used less frequently after the improvement of porous materials (11). We used a smooth polyamide sheet implant, SupraFOIL^®^ which was previously donated to our department. Stability was achieved by placing the implant over bony ledges to completely cover the fracture, and additionally making small holes in the implant to allow for orbital tissue ingrowth for long-term stability (9), which decreases complication rate to 1.7% (14).

Post-operative care may include systemic and topical antibiotics, as well as ice compresses and elevated head position to prevent and decrease edema. Visual acuity should be monitored regularly, along with pupillary size and reaction with globe motility anticipating signs of a retrobulbar hematoma. Late surgical complications may include eyelid deformations, diplopia, paresthesia in the area of the infraorbital nerve innervation, enophthalmos, and blindness (3). Diplopia is usually improved within a few weeks. Persistent diplopia is reported in a range of 8–42%, which indicates the importance of a forced duction test once the implant is inserted (15). Other reasons for diplopia include damage to the optic nerve, muscle, or fibrosis (1). Residual enophthalmos due to fat atrophy as a consequence of a late repair is found in a range of 7–27% (14). Additional surgery can be performed 3 months after the initial one using augmentation implants or fat tissue transplants (1). The main goals of orbital floor fracture repair are to reduce the herniating soft tissue and restore globe position, motility, and orbital volume.

This case report illustrates the surgical management of the orbital floor fracture from the aspects of oral-maxillofacial and oculoplastic surgeons. Although, there is substantial evidence in the literature that the smooth polyamide sheet implant material offers stabile and long-term solution for orbital fracture; to the best of our knowledge, it was the first case in Bosnia and Herzegovina to use this material and the transconjunctival approach for orbital floor repair.

## Disclosures

### Funding:

We confirm that there is no relationship with the commercial product smooth polyamide sheet implant, SupraFOIL^®^. No specific funding was received for this study.

### Informed consent:

Written informed consent was obtained from the patient for the publication of the case report and the accompanying images.

### Peer-review:

Externally peer-reviewed.

### Conflict of Interest:

None declared.

### Authorship Contributions:

Involved in design and conduct of the study (AP, NJ, EK, NB, NL, AK); preparation and review of the study (AP, NJ, NB, AK); data collection (AP, NJ, NB, NL); and language approval (NJ, AK).
